# New Appliances and Things Medical

**Published:** 1904-01-02

**Authors:** 


### NEW APPLIANCES AND THINGS MEDICAL.

### VERONAL.

(E. Merck, 16 Jewry Street, London, E.C.)

This preparation is a new synthetic hypnotic lately introduced from the Continent. Chemically it is di-ethylmalonylurea, and consists of small white crystals, odourless, and of slightly bitter taste. Its administration is said to be indicated in all cases of simple sleeplessness, and especially in conditions of neurasthenia, hypochondria, etc. As far as we have been able to judge, 5 grammes in the form of powder or tabloid will prove useful as an effective nervous sedative with no undesirable after effects. The statement that Veronal is a harmless drug very likely will be seen to be erroneous when further experience of its action has been noted. We should say that all hypnotics must be administered with care and under medical supervision, for probably none are devoid of some amount of danger.

The notice of Wall's fountain pen which appeared in our issue of the 19th ult., omitted to state where the pen might be obtained. The address of the maker is:—R. Clinton Hughes, 56 Gracechurch Street, London, E.C.

